# Use of antidepressants during pregnancy in the Netherlands: observational study into postpartum interventions

**DOI:** 10.1186/s12884-016-1184-5

**Published:** 2017-01-11

**Authors:** Noera Kieviet, Fokke de Jong, Fedde Scheele, Koert M. Dolman, Adriaan Honig

**Affiliations:** 1 Department of Pediatrics, Psychiatry Obstetrics Pediatrics Expert Center OLVG West, Jan Tooropstraat 164, 1061 AE Amsterdam, The Netherlands; 2 Department of Psychiatry, Psychiatry Obstetrics Pediatrics Expert Center OLVG West, Jan Tooropstraat 164, 1061 AE Amsterdam, The Netherlands; 3 Department of Gynaecology, Psychiatry Obstetrics Pediatrics Expert Center OLVG West, Jan Tooropstraat 164, 1061 AE Amsterdam, The Netherlands; 4 Department of Psychiatry, VU Medical Center, de Boelenlaan 1118, 1081 HZ Amsterdam, The Netherlands

**Keywords:** Pregnancy, Withdrawal, Observation, Finnegan, Depression

## Abstract

**Background:**

Psychiatric disorders and use of selective antidepressants during pregnancy can have negative effects on mother and infant postpartum. This study aimed to provide evidence-based recommendations on observation of antidepressant-exposed mother-infant dyads.

**Methods:**

In this observational study, mother-infant dyads were observed for possible consequences of either the maternal psychiatric disorder or fetal exposure to selective antidepressants during pregnancy. These possible complications can lead to medical interventions, including 1. adjustment of antidepressants 2. admission to the psychiatric department 3. additional investigations due to indistinctness about the origin of neonatal symptoms 4. treatment of poor neonatal adaptation and 5. consultation of an external organization for additional care. The type, number and time to medical interventions were analyzed.

**Results:**

In 61% of the 324 included mother-infant dyads one or more intrventions were performed. Adjustment of antidepressants and treatment of poor neonatal adaptation were most prevalent. In 75% of dyads the final intervention was performed within 48 h.

**Conclusions:**

The high prevalence and type of medical interventions requires professional observation of all mother-infant dyads exposed to selective antidepressants. In the absence of specialized home care, hospital admission is indicated whereby an observational period of 48 h seems sufficient for most dyads.

## Background

Exposure to selective antidepressants during pregnancy, used by 2–9% of Western pregnant women [1, 2], can have postpartum consequences for mother and infant [3]. In addition, the maternal psychiatric disorder itself can also have negative consequences during this period [4]. Therefore, postpartum hospital observation of selective antidepressant-exposed mothers and their infants is common practice [3, 5, 6]. Unfortunately there are no guidelines concerning the type and duration of this observational period.

Possible consequences of the maternal psychiatric disorder include an increased risk of prematurity and low birth weight and a recurrence or worsening of psychiatric symptoms, which can impair the attachment between mother and infant [4, 7–11]. Approximately 20–30% of selective antidepressant-exposed infants develop poor neonatal adaptation due to exposure to selective antidepressants [3, 12–14]. Poor neonatal adaptation consists of symptoms of restlessness, such as feeding and sleeping difficulties, which are mostly mild and self-limiting. Because symptoms are nonspecific, differentiation from other, more severe, neonatal pathology such as perinatal infection, can be difficult [3, 5, 13, 14]. Furthermore, some studies found that exposure to selective antidepressants may elevate the risk of other maternal and neonatal effects, such as postpartum hemorrhage, prematurity and persistent pulmonary hypertension of the newborn (PPHN). However other studies did not find a relation between exposure to selective antidepressant and these complications [15, 16].

Because the evaluation of possible maternal and neonatal effects need medical expertise or medical intervention, in the Netherlands, most caregivers advise hospital admission of mothers and their infants postpartum. However, the duration of this period differs per hospital.

Several experts advise an observational period of 48 h, though guidelines or protocols are not available [5, 12, 17–19]. Evidence-based recommendations on this subject would improve the quality and uniformity of care. In order to develop such recommendations, insight into complications that lead to medical intervention is needed.

This observational study provides insight into the type and number, as well as the time to medical interventions during the first days postpartum. From these data, recommendations can be formulated with respect to the necessity of professional observation, type of observational setting and time period of observation.

## Methods

### Setting

We conducted an observational study in a teaching hospital in Amsterdam, the Netherlands. The psychiatric, obstetric, pediatric clinic of this hospital is a center of expertise for pregnancy and psychiatric disorders and advises women with a psychiatric disorder before, during and after pregnancy. Primary care givers (mainly general practitioners, midwifes, obstetricians and psychiatrists) refer patients to this clinic. Approximately 50% of all women who visit this clinic live in the catchment area and deliver in our hospital. In case of home birth, or birth in another hospital due to capacity problems in our hospital, within 8hours postpartum these women are admitted to the maternity ward, together with their infants.

During hospital admission, nurses, pediatricians, obstetricians and psychiatrists observe, evaluate, support and treat mother and infant for 72 h. The psychiatrist evaluates possible psychiatric symptoms in mothers and adjusts the psychotropic medication if necessary (changes the dosage or type of psychotropic medication or starts additional medication). The psychiatrist uses the hospital anxiety and depression scale (HADS), which is a validated questionnaire for symptoms of anxiety and depression and is completed at the first day postpartum delivery [20, 21]. If psychiatric care after hospital discharge is too limited or in case social problems, such as absence of a support system, are detected external organizations can be consulted. The obstetrician assesses the physical condition of the mother and treats complications if necessary. Infants are physically examined by the pediatrician and treated in case of pathology. Trained nurses assess poor neonatal adaptation in infants with use of the Finnegan scoring list three times a day [22]. This observational tool is used to monitor the moment of onset and development of symptoms of poor neonatal adaptation. A Finnegan score of four or higher is an indication for poor neonatal adaptation [3, 5, 12]. The pediatrician determines if PNA has been present or absent, based on the Finnegan scores and physical examination and observation. In case of indistinctness about the origin of symptoms, additional testing can be necessary to exclude other neonatal pathology, such as perinatal infection [3, 5, 13, 14]. When poor neonatal adaptation has been diagnosed supportive measures such as swaddling are mostly sufficient. Infants with severe poor neonatal adaptation (multiple Finnegan scores of ≥ 8 and severe symptoms such as convulsions) are admitted to the neonatal care unit and can be treated with phenobarbital. Parents are supported by explanation and reassurance. Furthermore, the team observes the mother-infant interaction. If interaction difficulties are present external organizations can be consulted to provide family care.

### Participants

From January 1st 2007 to December 31st 2012 all mothers who used selective antidepressants during at least the last trimester of pregnancy and who were admitted in our hospital for observation together with their infants postpartum were included (*n* = 324). Selective antidepressants were defined as selective serotonin reuptake inhibitors, serotonin norepinephrine reuptake inhibitors, norepinephrine-dopamine reuptake inhibitors or noradrenergic and specific serotonergic antidepressants. Tricyclic antidepressants, were not included as these antidepressants have a different mechanism of action. However, if mothers used other types of antidepressants or other types of psychotropic drugs in combination with a selective antidepressants, mothers were included.

Exclusion criteria were use of illicit drugs [23] or regular alcohol use of more than two units per week during last trimester of pregnancy, admission to the psychiatric department up to delivery and postpartum transfer to another hospital. In order to create mother-infant dyads, in case of multiple pregnancies one infant was randomly selected.

### Interventions

Mother-infant dyads were analyzed for medical interventions that had been performed during hospital admission, all caused by exposure to selective antidepressants or the maternal psychiatric disorder. These interventions were: 1. adjustment of psychotropic medication 2. admission to the psychiatric department 3. additional investigations due to indistinctness about the origin of neonatal symptoms whereby the final diagnose was poor neonatal adaptation. 4. treatment of poor neonatal adaptation and 5. consultation of an external organization for additional family care. The prevalence of interventions was established by calculating the percentage of mother-infant dyads in which one or more interventions were performed. All base-line characteristics were compared between mother-infant dyads in which an intervention was performed and dyads whereby no intervention was performed.

### Time to interventions

For the first four interventions the time to intervention (hours postpartum) was analyzed. For the fifth intervention (consultation of an external organization for additional family care) time was not analyzed because organizations are generally contacted at hospital discharge.

Per mother-infant dyad the time to the final intervention was investigated. This was exclusively done for dyads in which times of all interventions were known.

The time to intervention was divided into <12 h, 13–24, 25–48, 49–72 and >72 h postpartum. The time to the intervention ‘treatment of poor neonatal adaptation’ was defined as the moment of administration of pharmacotherapy or the moment of the first Finnegan score of four or higher in case of supportive treatment. The time to other interventions was established by the time noted in the patient files of mother and infant.

In order to examine if there are factors which are associated with late time of intervention, all base-line characteristics were compared between mother-infant dyads in which the final intervention was performed within 48 h and dyads in which the final intervention was performed after 48 h postpartum.

### Statistical analyses

Data were analyzed by SPSS version 21 (IBM, New York, USA). In all continuous variables, the mean and standard deviation were presented in case of a normal distribution. Otherwise, the median and inter quartile range were presented. The base-line characteristics of mother-infant dyads in which an intervention was performed were compared to the characteristics of dyads in which no intervention was performed. Continuous, normally distributed variables were compared with the independent sample *t* test. Continuous, skewed variables were compared with the Mann Whitney *U* test. Dichotomous or categorical variables were compared with the chi square test. In case more than 20% of the expected cell counts were less than five the Fisher exact test was performed. A result was considered statistically significant if the *p*-value was not larger than 0.05. The base-line characteristics of dyads in which the final intervention was performed within or after 48 h were compared in the same manner. In addition, odds ratios (OR) and 95% confidence intervals (CI) were estimated using univariate analysis.

## Results

### Patient characteristics

**Fig. 1:**
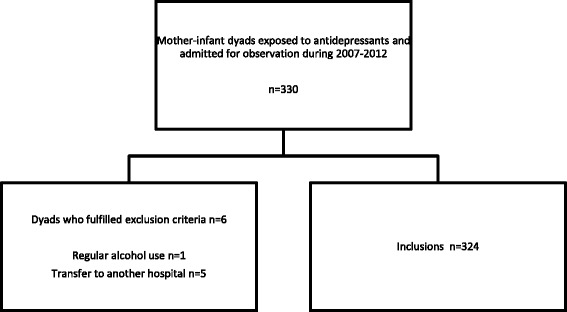
Flow chart of inclusions and exclusions of mother-infant dyads

In the period of January 1st 2007 till December 31st 2012, a total of 330 mothers who used selective antidepressants during at least the last trimester of pregnancy were admitted to the maternity ward of our hospital for observation. Two mothers delivered a twin, one of each twin was randomly excluded. One mother-infant dyad was excluded because the mother used soft drugs during pregnancy and five dyads were excluded because they were transferred to another hospital. In three dyads this transfer was based on severe medical problems, which were fluxus, neonatal breathing difficulties and neonatal asphyxia. In the end, 324 mother-infant dyads (98.2%) were included (Fig. 1). Of these mothers, 304 mothers (93.8%) delivered in our hospital, 13 mothers (4.0%) delivered at home and 7 mothers (2.2%) delivered in another hospital. The maternal and neonatal characteristics are presented in Table 1. Three mothers (0.9%) decided to go home before medical discharge, all after at least 48 h of observation. Of the 324 mothers, 310 (95.7%) were known with an affective disorder, some in combination with other psychiatric disorders. Of the 291 mothers who solely used an antidepressant, 209 used an selective serotonin reuptake inhibitors, 32 a serotonin norepinephrine reuptake inhibitors, 35 a noradrenergic and specific serotonergic antidepressants and 1 a norepinephrine-dopamine reuptake inhibitor. Fourteen mothers used a combination of antidepressants. See Appendix for an overview of antidepressants used in our study population.

**Table 1:** Characteristics of mothers and infants

Variables	Total group *n* = 324	Intervention *n* = 143	No intervention *n* = 181	*p*-value
	Median (inter quartile range)			
Maternal age during delivery (years)	33 (29–36)	32 (29–36)	33 (29–36)	0.59
Duration of observation mother (days)	4 (3–5)	4 (3–6)	4 (3–5)	0.28
Duration of observation infant (days)	4 (3–5)	4 (3–5)	4 (3–5)	0.14
	n (%)			
Marital status				0.80
Married/living together	275 (85.9)	121 (85.2)	154 (86.5)	
Living Apart Together	17 (5.3)	7 (4.9)	10 (5.6)	
Single	28 (8.8)	14 (9.9)	14 (7.9)	
Unknown	4	1	3	
Smoking	31 (9.8)	16 (11.6)	15 (8.5)	0.36
Unknown	9	5	4	
Type of psychotropic medication				0.37
Solely antidepressant	291 (89.8)	126 (88.1)	165 (91.2)	
Antidepressant and other type of psychotropic drug	33 (10.2)	17 (11.9)	16 (8.8)	
Dosage of antidepressant				0.003
Below minimal effective daily dosage	20 (6.2)	8 (5.6)	12 (6.6)	
Minimal effective daily dosage	146 (45.2)	50 (35.2)	96 (53.0)	
Above minimal effective daily dosage	157 (48.6)	84 (59.2)	73 (40.3)	
Unknown	1			
Primiparous	144 (44.4)	65 (45.5)	79 (43.6)	0.75
HADS^a^				
Anxiety scale elevated	131 (42.5)	75 (55.1)	67 (39.0)	0.02
Depression scale elevated	93 (30.2)	68 (50.0)	63 (36.6)	0.01
One or both scales elevated	142 (46.1)	75 (55.1)	67 (39.0)	0.01
Unknown	16	7	9	
Gender infant male	163 (50.3)	78 (54.5)	85 (47.0)	0.18
Type of birth				0.07
Vaginal, non-instrumental	236 (72.8)	97 (67.8)	139 (76.8)	
Vaginal, instrumental	37 (11.4)	16 (11.2)	21 (11.6)	
Caesarean Section	51 (15.7)	30 (21.0)	21 (11.6)	
Type of feeding				0.02
Breastfeeding or mixed feeding	250 (77.4)	101 (71.1)	149 (82.3)	
Exclusively formula	73 (22.6)	41 (28.9)	32 (17.7)	
Unknown	1			
Prematurity	30 (9.3)	13 (7.0)	17 (9.4)	0.93
Finnegan score, highest during observation				<0.001
< 4	130 (40.1)	30 (21.0)	100 (55.2)	
4–8	156 (48.1)	80 (55.9)	76 (42.0)	
≥ 8	38 (11.7)	33 (23.1)	5 (2.8)	

^a^Hospital anxiety and depression scale

### Interventions

Of the 324 mother-infant dyads, a total of 143 dyads (61.1%) needed one or more intervention. One intervention was performed in 70.6% of dyads. In 24.5% two and in 4.9% three or four interventions were performed.

In 76 mothers (23.5%) the psychotropic medication was adjusted. The main reasons were symptoms of depression or anxiety and sleeping difficulties. No mothers had to be admitted to the psychiatric department. Nine infants (2.8%) underwent additional tests due to indistinctness about the origin of neonatal symptoms. Additional testing, which included blood tests, brain ultrasounds, a chest radiograph and electroencephalography excluded other pathology where after poor neonatal adaptation was diagnosed in all nine infants. The pediatrician diagnosed poor neonatal adaptation in 76 infants (23.5%). These infants showed mild symptoms of sleeping- as well as feeding difficulties, tremors and jitteriness. One infant was treated with phenobarbital as these symptoms of restlessness were more severe and prolonged. All other infants were treated with supportive measures. External support was organized for eight mother-infant dyads (2.5%), which consisted of psychiatric home care, social welfare and support regarding infant development.

### Time to interventions

**Fig. 2:**
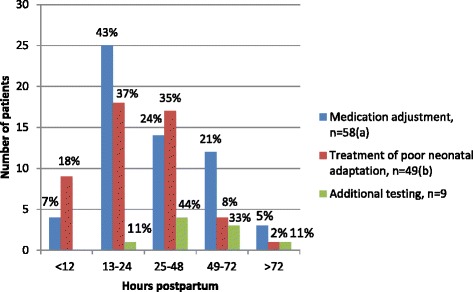
Time to interventions. a. Data on the time of medication adjustment were missing in 18 mother-infant dyads. b. Data on the time of treatment of poor neonatal adaptation were missing in 27 mother-infant dyads

The time to interventions are presented in Fig. 2. In 63 of the 143 dyads in which an intervention was performed, the time of at least one intervention was unknown. Thus, we analyzed time to the final intervention in 80 mother-infant dyads. In 67.5% of these dyads one intervention was performed and in 32.5% multiple interventions were performed. In 6 of the dyads (7.5%), the final intervention was performed within 12 h, in 28 (35%) between 13 and 24 h, in 26 (32.5%) between 25 and 48 h, in 16 (20.0%) between 49 and 72 and in 4 (5.0%) after 72 h postpartum.

Base-line characteristics were compared between mother-infant dyads in which the final intervention was performed within 48 h and dyads in which the final intervention was performed after 48 h postpartum (Table 2). This revealed no statistically significant differences.

**Table 2:** Comparison of base-line characteristics between mother-infant dyads whereby the final intervention was performed within 48 h and after 48 h postpartum

Variables	Intervention <48 h *n* = 60	Intervention >48 h *n* = 20		*p*-value
	Mean (standard deviation)			
Maternal age during delivery (years)	33.3 (4.7)	31.9 (4.3)		0.24
	Median (interquartile range)			
Duration of observation mother (days)	4 (3–5)	4 (3.3–5)		0.68
Duration of observation infant (days)	4 (3–5)	4 (3.3–5)		0.79
	n (%)		Odds ratio 95% CI	
Marital status				0.26*
Single	6 (60.0)	4 (40.0)	1 (ref)	
Married/living together/LAT^a^	54 (77.1)	16 (22.9)	0.44 0.11–1.77	
Smoking				1.00*
No	55 (76.4)	17 (23.6)	1 (ref)	
Yes	5 (83.3)	1 (16.7)	0.65 0.07–5.93	
Unknown	0	2		
Type of psychotropic medication				1.00*
Solely antidepressant	54 (75.0)	18 (25.0)	1 (ref)	
Antidepressant and other type of psychotropic drug	6 (75.0)	2 (25.0)	1.00 0.19–5.40	
Dosage				0.19
Minimal or below minimal effective daily dosage	25 (83.3)	5 (16.7)	1 (ref)	
Above minimal effective daily dosage	35 (70.0)	15 (30.0)	0.47 0.15–0.69	
Parity				0.16
Primiparous	32 (82.1)	7 (17.9)	1 (ref)	
Multiparous	28 (68.3)	13 (31.7)	2.12 0.74–6.06	
HADS^b^				
Anxiety scale not elevated	27 (73.0)	10 (27.0)	1 (ref)	0.74
Anxiety scale elevated	32 (76.2)	10 (23.8)	0.84 0.31–2.33	
Depression scale not elevated	32 (74.4)	11 (25.6)	1 (ref)	0.95
Depression scale elevated	27 (75.0)	9 (25.0)	0.97 0.35–2.69	
No scales elevated	24 (75.0)	8 (25.0)	1 (ref)	0.96
One or both scales elevated	35 (74.5)	12 (25.5)	1.03 0.37–2.89	
Unknown	1	0		
Gender infant				0.60
Male	32 (72.7)	12 (27.3)	1 (ref)	
Female	28 (77.8)	8 (22.2)	0.76 0.27–2.13	
Type of birth				0.66
Vaginal	45 (76.3)	14 (23.7)	1 (ref)	
Caesarean Section	15 (71.4)	6 (28.6)	1.29 0.42–3.94	
Type of feeding				0.67
Breastfeeding or mixed feeding	42 (73.7)	15 (26.3)	1 (ref)	
Exclusively formula	18 (78.2)	5 (21.7)	0.78 0.25–2.46	
Gestational age				0.57*
< 37 weeks	4 (100.0)	0 (0.0)	Not possible to calculate	
≥ 37 weeks	56 (73.7)	20 (26.3)		
Finnegan score, highest during observation				0.50*
< 4	12 (85.7)	2 (14.3)	1 (ref)	
≥ 4	48 (72.7)	18 (27.3)	2.25 0.46–11.06	

Odds ratios and 95% confidence intervals are presented

*Calculated with Fisher exact test

^a^LAT: Living apart together

^b^HADS: Hospital anxiety and depression scale

## Discussion

To the best of our knowledge, this is the first study presenting data on medical interventions during the course of postpartum observation of mother-infant dyads exposed to selective antidepressants during pregnancy. In 61% of all dyads one or more interventions were performed. The most prevalent interventions were adjustment of psychotropic medication and treatment of poor neonatal adaptation. As expected, the Finnegan scores of infants in the group whereby interventions were performed were higher (*p* = <0.001) and the level of symptoms of anxiety and depression was higher in the intervention group. Also, in mother-infant dyads whereby an intervention was performed, mothers used a relatively higher antidepressant dosage. The type of underlying psychiatric disorder and severity of psychiatric symptoms might attribute to this relationship. PNA does not seem to be related to the antidepressant dosage [13, 24]. In mother-infant dyads whereof the infants were formula-fed, the intervention rate was higher. An earlier study of our research group showed that formula-fed infants develop more symptoms of poor neonatal adaptation, which could explain this finding [24]. However, other factors, such as the type of psychotropic drugs (some are not compatible with breastfeeding) and severity of psychiatric disorder might also attribute to this relationship. The other base-line characteristics did not differ significantly.

Given the high prevalence of interventions, we would advise to observe all selective antidepressant-exposed mother-infant dyads. Thereby, the observational setting is a matter of debate. There are several disadvantages of hospital admission, including the risk of infection and lack of privacy. However, interventions that were most prevalent; adjustment of the psychotropic medication and establishment of poor neonatal adaptation, have to be performed by professionals [5, 25]. In the absence of specialized home care in a country, hospital admission is indicated.

Based on our results, we would advise to observe mother-infant dyads for 48 h. This advice is based on our finding that the final intervention was performed within 48 h in the majority of dyads (75%), while after an observational period of 24 h this percentage was only 43%. However, in 25% of mother-infant dyads the final intervention was performed after 48 h postpartum. Base-line characteristics of this group of dyads did not differ from base-line characteristics of dyads in which the final intervention was performed within 48 h postpartum.

It is especially difficult to determine the duration of the observational period for mother-infant dyads with no other indication for hospital admission apart from the exposure to selective antidepressants. Therefore, we performed a post-hoc analysis whereby we excluded dyads with an additional indication for hospital admission directly postpartum, such as a caesarean section or prematurity (*n* = 165). Of the remaining 159 dyads, 60 (37.7%) needed one or more intervention. The prevalence of interventions was higher in the group of dyads with an additional indication for hospital admission compared to the group of dyads whereby selective antidepressant-exposure was the only indication for hospital admission (p 0.02). A possible explanation for this finding might be that indications for hospital stay, such as prematurity or fluxus might be associated with selective antidepressant exposure [15, 26].

By interpretation of our results, it is important to hold into account that the percentage of mother-infant dyads whereby an intervention was performed is based on 5 types of neonatal and maternal interventions which differ in type and underlying medical problem. This also accounts for the time to interventions, whereby 4 interventions were analyzed.

This study has several limitations. First of all, there were missing data due to the observational design of this study. However, there is no indication that these data were selective. Therefore the reliability of our results is not likely to be affected. Furthermore, hospital admission entails the risk of over diagnosis; some observed effects, which resulted in additional testing or therapy, would not have been observed nor have led to problems in the home setting. Thereby a longer observational period might result in a higher risk of over diagnosis. Apart from the detriment for mother and infant, this may also have affected our study results by increasing the prevalence of interventions. Also, it is important to realize, that this study was performed in a center of expertise for pregnancy and psychiatry. All women were evaluated by a psychiatrist and all infants by a pediatrician. This thorough examination may have resulted in an accurate prevalence. However it is plausible that the prevalence of interventions in this study is higher compared to centers without this specialized care. Furthermore, mother-infant dyads which had been transferred to another hospital were excluded because there was no or a limited observational period in our hospital. As this transfer was based on severe maternal or neonatal pathology in three mother-infant dyads, this may have lead to a relative underestimation of the prevalence of interventions.

## Conclusions

In conclusion, in mother-infant dyads exposed to selective antidepressants during pregnancy, the prevalence of medical interventions was 61%. The most prevalent interventions were adjustment of psychotropic medication and treatment of poor neonatal adaptation. The high prevalence and type of medical interventions requires professional observation of all mother-infant dyads by a multidisciplinary team including a psychiatrist, pediatrician and trained nurses. In the absence of specialized home care, hospital admission is indicated. An observational period of 48 h seems sufficient for most mother-infant dyads. Further studies that examine predictors of intervention during observation or predictors of a prolonged observation, would be of additional value. If these predictors are known, it might be possible to provide an advise regarding the type and duration of observation on individual basis. In addition, a cost-effectiveness analysis would also be essential for development of a guideline.

## Appendix

**Table 3:** Overview of antidepressants used in our study population

	Number of patients (total 324)
Paroxetine	58
Paroxetine and mirtazapine	3
Paroxetine and olanzapine	2
Paroxetine and risperidone	1
Sertraline	71
Sertraline and mitrazapine	3
Sertraline and haloperidol	3
Sertraline and quetiapine	1
Sertraline and flupentixol	1
Fluoxetine	18
Fluoxetine and mirtazapine	1
Fluoxetine and quetiapine	1
Citalopram	58
Citalopram and mirtazapine	4
Citalopram and amitryptiline	1
Citalopram, amitriptyline and mirtazapine	1
Citalopram and olanzapine	1
Citalopram and pipamperone	1
Citalopram and haloperidol	1
Citalopram and lithium	1
Citalopram and oxaxepam	1
Venlafaxine	32
Venlafaxine, mirtazapine and haloperidol	1
Venlafaxine and haloperidol	1
Venlafaxine and quetiapine	5
Venlafaxine and olanzapine	1
Fluvoxamine	4
Fluvoxamine and lithium	1
Mirtazapine	35
Mirtazapine and clompiramine	1
Mirtazapine and haloperidol	4
Mirtazapine and risperidone	2
Mirtazapine and quetiapine	1
Mirtazapine and perfenazine	1
Mirtazapine and flurazepam	1
Bupropion	1
Bupropion and quetiapine	1

## Abbreviations

CI: Confidence interval
HADS: Hospital anxiety and depression scale
LAT: Living apart together
OR: Odds ratio
PPHN: Persistent pulmonary hypertension of the newborn
